# A Method for Preventing Aerosols During Dental Treatment With an Oroscope

**DOI:** 10.7759/cureus.22896

**Published:** 2022-03-06

**Authors:** Yuki Kojima, Ryozo Sendo

**Affiliations:** 1 Anesthesiology, Asahi General Hospital, Asahi, JPN; 2 Anesthesiology, Imakiire General Hospital, Kagoshima, JPN

**Keywords:** dental treatment, oroscope, infection, wuhan coronavirus, aerosol

## Abstract

Dentists have a much higher risk of exposure to coronavirus disease (COVID-19) than other healthcare workers. The virus is transmitted primarily through respiratory droplets and close/direct contact. Aerosol propagation is also possible in the case of prolonged exposure to high concentrations in a relatively closed environment. In this report, we describe the use of an aerosol box model to prevent aerosol generation during dental procedures. This report serves to inform clinicians on the potential effectiveness of this stopgap measure in cases where aerosol-generating procedures are unavoidable and medical supplies and personal protective equipment are in short supply.

## Introduction

Coronavirus disease (COVID-19) has been declared a global pandemic of international emergency. Its spread is especially difficult to manage given that infected individuals may be asymptomatic [1]. The virus is transmitted primarily through respiratory droplets and close/direct contact. Aerosol propagation is also possible in the case of prolonged exposure to high concentrations in a relatively closed environment [2]. Once generated, aerosols remain suspended in the air for approximately three hours. On March 15, 2020, the New York Times published an article entitled “The Workers Who Face the Greatest Coronavirus Risk,” in which a schematic figure demonstrated that dentists have a much higher risk of exposure to COVID-19 than other healthcare workers, such as nurses and general physicians [3]. This is because dental treatment is often performed in a closed space, requiring the dentist to be close to the patient. Furthermore, it has been established that severe acute respiratory syndrome coronavirus 2 (SARS-CoV-2) may be present in the saliva of asymptomatic individuals, suggesting that aerosol-generating dental procedures can be a source of infection [4].

Infection of dentists is likely to cause secondary infections in non-infected individuals. The inability to receive adequate dental care has many adverse effects on general health. Therefore, it is essential to ensure that satisfactory measures are in place to facilitate the safe and effective delivery of dental care. It is critical that such care is not in itself a source of infection to protect public health.

In this report, we described a new technique for infection prevention during dental treatment. This technique involves an aerosol box and endoscope. We proposed that the new technique would be effective for routine clinical work after the pandemic.

## Technical report

Researchers have recently reported the concept of using an aerosol box to prevent aerosol generation during intubation [5]. Subsequent modifications have produced more convenient and practical devices [6]. Using this aerosol box model, we devised a method to suppress aerosol generation by using a surgical endoscope adapted for oral examination, called an “oroscope” (Video 1).

**Video 1:** Methods of technique.

**Figure 1:**
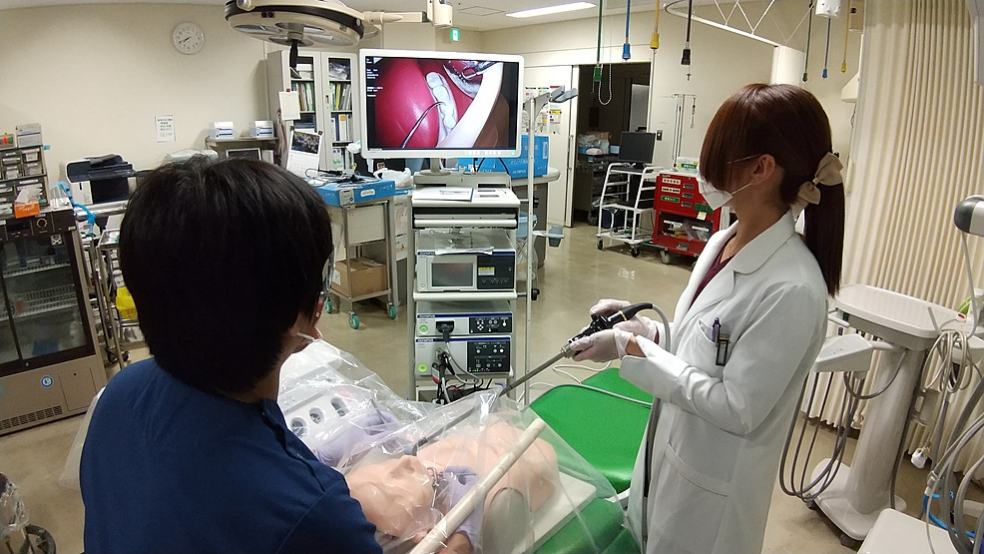
Using "oroscope" technique. The simulated patient is covered with a plastic bag. As a result, any aerosols generated by the dental procedures remain in the plastic bag. The field of view can be secured by using an “oroscope.”

The dentist places a plastic bag over the chest and face of the patient, who is seated on the dental chair, and the dental instruments to be used in the procedure. As a result, any aerosols generated by the dental procedures remain in the plastic bag. This will prevent the infection of dentists and dental hygienists and reduce the contamination of the clinical environment. However, the deposition of aerosol particles onto the inner surface of the plastic bag results in a gradual deterioration of the surgical field of view. Therefore, the “oroscope” is used to secure the field of view. In the present case, we inserted an endoscope (LTF-s190-10, Olympus, Japan), specifically designed for laparoscopy, through a small opening in the plastic bag, limiting the transmission of aerosols (Figure 1). We have termed this infection prevention approach the “Kojima/Sugimura dental treatment system” (KS system).

## Discussion

Verification of the effectiveness of this device in containing all aerosols generated by typical dental procedures is pending. However, it appears to be effective as a stopgap measure in cases where aerosol-generating procedures are unavoidable and medical supplies and personal protective equipment are in short supply. It can be effective in areas where personal protective equipment is scarce. Dentists may use an “oroscope” for patients undergoing any type of dental procedure; this would be especially beneficial in cases where infection status is uncertain. Some patients may find the placement of the vinyl cover over their face to be annoying. However, there is no immediate harm to the patient. As dentists will usually be focused on the visual display of the “oroscope,” which provides a narrow field of view, they may need to regularly shift their focus to assess the larger operative field. Previous studies have suggested that videoscopes may be effective for periodontal surgery and implant therapy, and no major complications have been reported [7,8].

While the concept of using a ventilation system to remove aerosols that are generated during dental procedures has been recently proposed, such a system would have potential drawbacks, including difficulties with large-scale manufacturing and the inability to completely prevent infection transmission [9]. The endoscope used in this report was based on a previously established model and adapted for use in the oral cavity and pharynx. We have not experienced any problems with its use, and it appears to provide a sufficient field of view for routine dental treatment. As aerosols cannot be transmitted through the vinyl cover, the proposed device can theoretically contain the aerosols generated by infected patients. The clinical effectiveness of the “KS system” requires confirmation by additional studies.

Given the current lack of a robust COVID-19 vaccine, the only viable option for health care providers is to prevent the transmission of the disease from infected individuals. Since aerosols are often generated during dental procedures, the “KS system” may be able to significantly reduce COVID-19 spread to dental healthcare professionals. Even after a permanent vaccine and treatment for COVID-19 are established, the use of an “oroscope” may remain essential to prevent other forms of aerosol infections; furthermore, it may also reduce the consumption of personal protective equipment by healthcare workers.

In the context of the current COVID-19 pandemic, dental treatment is stressful for both patients and dental healthcare professionals [10-12]. By using the “KS system,” dentists may lower the transmission of aerosol infections and reduce the stress and anxiety of potentially contracting an infection during dental procedures. This method can be applied even after the pandemic and used as one of the infection control methods in the future. Moreover, an oroscope may be effective for securing the visual field during the treatment of molar teeth, which is difficult to treat. It is necessary to continue to examine how effective this method is.

## Conclusions

We devised a method to prevent aerosol infection and contamination of the clinical environment in dentistry practices by using an oroscope and a plastic bag placed over the chest and face of the patient. This method can be applied even after the pandemic and may be used as one of the infection control methods in the future. Furthermore, an oroscope may be effective for securing the visual field.
